# A case of musical preference for Johnny Cash following deep brain stimulation of the nucleus accumbens

**DOI:** 10.3389/fnbeh.2014.00152

**Published:** 2014-05-06

**Authors:** Mariska Mantione, Martijn Figee, Damiaan Denys

**Affiliations:** ^1^Department of Psychiatry, Academic Medical Center, University of AmsterdamAmsterdam, Netherlands; ^2^Netherlands Institute for Neuroscience, an Institute of the Royal Netherlands Academy of Arts and SciencesAmsterdam, Netherlands

**Keywords:** nucleus accumbens, deep brain stimulation, obsessive-compulsive disorder, musical preference, reward system

## Abstract

Music is among all cultures an important part of the live of most people. Music has psychological benefits and may generate strong emotional and physiological responses. Recently, neuroscientists have discovered that music influences the reward circuit of the nucleus accumbens (NAcc), even when no explicit reward is present. In this clinical case study, we describe a 60-year old patient who developed a sudden and distinct musical preference for Johnny Cash following deep brain stimulation (DBS) targeted at the NAcc. This case report substantiates the assumption that the NAcc is involved in musical preference, based on the observation of direct stimulation of the accumbens with DBS. It also shows that accumbens DBS can change musical preference without habituation of its rewarding properties.

## Introduction

Mr. B., a 59-year old married man, was referred to the department of Anxiety disorders, suffering from obsessive-compulsive disorder (OCD) for 46 years. He reported obsessions about fear for uncertainty and illogical things, and compulsions about seeking reassurance and hoarding. At the moment of referral, Mr. B. scored a total of 33 points on the Yale-Brown obsessive-compulsive scale (Y-BOCS; Goodman et al., 1989a,b), corresponding with extremely severe OCD. He scored 18 points on the Hamilton Anxiety Scale (HAM-A; Hamilton, 1959), corresponding with moderate anxiety and 14 points on the Hamilton Depression Scale (HAM-D; Hamilton, 1960), corresponding with mild depression. In spite of extensive treatment with pharmacotherapy and cognitive behavioral therapy, symptoms were still overpowering and Mr. B. remained extremely hindered in daily living.

Suffering from treatment-refractory OCD, Mr. B., was included for treatment with deep brain stimulation (DBS) targeted at the nucleus accumbens (NAcc). After signing informed consent, Mr. B., was implanted in December 2006 with two four-contact electrodes (Model 3389, Medtronics, Minneapolis). The electrodes were connected via subcutaneous extensions to Soletra stimulators (Medtronic, Minneapolis) placed bilaterally in an infraclavicular pocket under general anesthesia. The center of the deepest contacts was positioned 3 mm in front of the anterior border of the anterior commissure (AC), 7 mm lateral and 4 mm below the line connecting the anterior and posterior commissure (PC). This was verified by post-operative CT, fused with preoperative MR.

After DBS surgery Mr. B. entered an optimization phase in which optimal stimulation parameters were adjusted. Within 6 weeks after surgery Mr. B. experienced a decline in anxiety and obsessions, with stimulation parameters fixed on contacts 2 and 3 set negative and case set positive, a voltage of 5.0 V., a pulse width of 90 ms and a frequency of 185 Hz. It was notable that, for the first in years, he was not seized by panic and able to postpone his compulsions. After this initial decrease in obsessive-compulsive symptoms a standardized cognitive behavioral treatment program was added to address his avoidant behavior. Within 6 months his symptoms decreased gradually to a Y-BOCS of 8 points, corresponding with mild OCD. The HAM-A declined to 4 points, corresponding with subclinical anxiety and the HAM-D declined to 2 points, corresponding with subclinical depression. Mr. B. reported he felt very confident, calm and assertive and he started to call himself “Mr. B. II”, the new and improved version of himself.

Mr. B., had never been a huge music lover. His musical taste was broad, covering Dutch-language songs, the Beatles and the Rolling Stones, with a preference for the last named. While music did not occupy an important position in his live, his taste in music had always been very fixed and his preferences stayed the same throughout decades. On average, a half year after DBS surgery, Mr. B. stated that he was turning into a Johnny Cash fan. He had been listening to the radio, when he coincidentally heard “Ring of Fire” of the Country and Western singer and experienced that he was deeply affected by the song. Mr. B. started to listen to more songs of Johnny Cash and noticed that he was deeply moved by the raw and low-pitched voice of the singer. Moreover, he experienced that he preferred the performance of the songs in the Seventies and Eighties, due to the fullness of the voice of the older Johnny Cash in that period. His favorite songs, “Folsom prison blues”, “Ring of fire” and “Sunday morning coming down” had a certain rhythm with a fast tempo in common that moved him. Mr. B. reported that he felt good following treatment with DBS and that the songs of Johnny Cash made him feel even better. From this moment on, Mr. B. kept listening simply and solely to Johnny Cash and bought all his CD’s and DVD’s. When listening to his favorite songs he walks back and forth through the room and feels like he finds himself in a movie in which he plays the hero’s part. He reports that there is a Johnny Cash song for every emotion and every situation, feeling happy or feeling sad and although Mr. B. played almost simply and solely Johnny Cash songs for the following years, the music never starts to annoy him. From the first time Mr. B. heard a Johnny Cash song, the Dutch-language songs, the Beatles and the Rolling Stones have been banned. Except when the stimulators run down or accidentally go out. Then, Johnny Cash is unconsciously ignored and his old favorites are played once again, just as it was for the past 40 years.

## Background

Most people find music to be an important part of their lives, whatever culture they may be from. Research on how the brain processes music is emerging. It appears that the auditory cortical regions contain specializations for analyzing and encoding pitch-based (melodic) relations and time-based (rhythmic) relations in music. Interactions between auditory areas and the frontal cortices, via ventral and dorsal routes, are crucial in allowing working memory to bring these elements of music together in abstract representations. These representations produce tonal and temporal expectancies based on structural regularities found in music (Zatorre and Salimpoor, 2013). There appears to be an implicit and generalized knowledge of musical rules and regularities due to exposure to music of a given genre. Additionally, there is more explicit knowledge when listeners are familiar with a specific piece of music and anticipation arises because they know that a particular event is followed by another. Depending on whether expectancies are violated or confirmed, listeners can experience tension and suspense or relaxation. The resulting moments of anticipation and resolution are believed to be a basis for emotions and rewarding experiences in response to music (van den Bosch et al., 2013). Since the pleasurable aspects of music differ from the functional characteristics of other reward stimuli, it has been suggested that the rewarding properties of music are realized indirectly by influencing these emotions. Musical taste, i.e., whether a particular piece of music is experienced as rewarding, may thus depend on individually defined cortical representations of the structure of this music, in interaction with brain systems involved in emotions and rewards. Indeed, our favorite songs may evoke strong rewarding emotions (Sloboda and Juslin, 2001), and even when music is unfamiliar to us and heard without a conscious goal, it can elicit strongly positive feelings (Brown et al., 2004) accompanied by physical responses (Panksepp, 1995) and this may depend on interactions of the cortical system with the striatal dopaminergic system (Zatorre and Salimpoor, 2013).

However, favorite songs and musical styles vary widely among people and various individual factors, such as personality, self-esteem, age, sex and income have been addressed to a greater or lesser extent as sources of variation in musical taste (North, 2010). As of yet, no anatomical or biological substrate has been identified that is explicitly related to musical preference. The current case report may provide important insights into the neural correlates of musical preference, by suggesting a relationship between neural stimulation and taste of music.

## Discussion

This exceptional case shows a patient who developed a distinct and entirely novel musical preference for Johnny Cash accompanied by powerful positive feelings, that was present during stimulation of the accumbens but absent during off stimulation. There are two remarkable aspects in this clinical observation that may suggest an association between DBS and changed musical preference. Firstly, preference for musical styles usually develops during late adolescence and early adulthood, and tends to prevail for the rest of people’s lives (Holbrook and Schindler, 1989). The patient, on the contrary, developed a sudden change in musical taste at 60 years of age. Moreover, his former musical taste reoccurred immediately when stimulation was interrupted due to battery depletion, suggesting a direct causal link between musical preference and stimulation of the accumbens. Secondly, while music formerly did not play an important role in his life, following stimulation music became suddenly extremely rewarding to the patient. Contrary to our normal experiences where repetitive listening to the same music or song eventually results in a habituation to its rewarding properties, in this case, the Johnny Cash songs never started to annoy the patient and kept the enduring capacities of pleasure and reward.

Our case appears to substantiate the idea that the NAcc plays a fundamental role in the rewarding properties of music. It has been proved that the ventral striatum and in particular the NAcc is a central region for processing reward and pleasure information. Increases in NAcc neuron activity and dopamine release are observed during expectations and experience of rewards (de la Fuente-Fernández et al., 2002; Adinoff, 2004; Schultz, 2004; Doyon et al., 2005). Even behavioral addictions that are non-drug related turn out to alter the reward circuit, consisting of dopamine projections linking the ventral tegmental area, NAcc, and part of the frontal lobe (Holden, 2001). Increases in ventral striatal activity have been associated with the rewarding properties of food consumption (Volkow and O’Brien, 2007), cocaine-induced euphoria (Breiter et al., 1997), monetary reward (Knutson et al., 2001a; O’Doherty et al., 2001) and nicotine addiction (Brody, 2006). Even when no explicit reward is present, as may be the case with listening to pleasant music, brain reward structures appear to be involved (Blood and Zatorre, 2001; Menon and Levitin, 2005; Koelsch et al., 2006). Menon and Levitin (2005), for example, have shown that passive listening to music results in a significant activation of subcortical structures including the NAcc. Salimpoor et al. (2011) have demonstrated that intense pleasure in response to music was associated with dopamine release in the ventral striatum. It is likely that musical NAcc activation directly increases hypothalamic and insula regions, which are thought to regulate physiological responses to rewarding stimuli (Menon and Levitin, 2005).

We recently showed that DBS of the ventral striatum in OCD restores neural processing of rewarding stimuli (Figee et al., 2013), explaining how NAcc DBS may influence the sensitivity to natural rewards such as music. Moreover, NAcc DBS in the current case was also associated with the kind of music that was preferred. In agreement, Salimpoor et al. (2013) demonstrated that the NAcc was activated when listeners heard their selected pleasurable music. Notably, it has been suggested that musical preference is encoded by the NAcc through interaction with cortical regions as a function of previous experiences with musical sounds. The current observations may imply however, that the NAcc could encode musical preference by itself and without any previous experience with the particular type of music. Alternatively, DBS may alter musical preference by modulation of corticostriatal networks. The frontostriatal reward circuitry is activated during highly pleasurable experience of music (Blood and Zatorre, 2001), especially during affective processing of music (Khalfa et al., 2005) and when music is personally preferred and familiar (Pereira et al., 2011). Recently, our group demonstrated that DBS is able to down-regulate excessive functional network connectivity in OCD, whereas listening to music increases functional network connectivity (Wu et al., 2012). Although highly speculative, listening to music may serve as a new and healthier way to engage brain networks following DBS-induced remission of compulsive behaviors. It has been suggested that the cortical system is able to predict tonal or rhythmic relationships in music, which are experienced as pleasurable via interaction with the ventral striatum (Zatorre and Salimpoor, 2013). Our patient reported that after DBS he was grasped in particular by the rhythm of Johnny Cash songs and by the tone of his voice. Hence, DBS may have changed musical preference by modulating frontostriatal decoding of tonal and rhythmic relationships.

One may, of course, argue that during stimulation the patient developed a new kind of obsession and compulsion while his former obsessions of fear for uncertainty and illogical things declined. His preference for Johnny Cash, however, does not match the definition of obsessions or compulsions. Listening to Johnny Cash is pleasurable and not preceded by anxiety, nor is discomfort provoked when the patient is prevented from listening. The patient does not feel obsessed with Johnny Cash, nor compelled to listen and his behavior does not result in reduction of anxiety or tension. Alternatively, DBS may have changed musical preference by influencing self-confidence. In contrast with the patients’ life before surgery, having far less OCD, anxiety and depressive symptoms, he felt highly confident after DBS, characterizing himself as “Mr. B. II”. It could be suggested that the image he creates when listening to songs of Johnny Cash seems to match his “new” confident self. Like Mr. B. states: “it seems as if Johnny Cash goes together with DBS”. Eventually, the strong feelings of pleasure that this self-created image elicits, implies that the associated behavior is rewarding and is likely to be repeated (Sloboda and Juslin, 2001; Huron, 2006). It has been shown that self-esteem influences musical preference, although it does not explain the largest part of the relationship between individual factors and musical taste. Besides, it appears that one of the reasons for listening to a favorite musical style for men is to use imagination and to create an image of oneself (North, 2010). Together with the aspect that music is rewarding due to the emotions it enhances (Salimpoor et al., 2009), both prefacing factors may have influenced the development of a distinct musical preference for Johnny Cash in our patient.

## Concluding remarks

Together these findings demonstrates involvement of the NAcc in the rewarding properties of music and suggests that DBS may alter musical preference by modulation of broader networks. Besides, it shows that accumbens DBS may change musical preference without habituation of its rewarding properties. However, more research is needed to confirm our preliminary findings and to understand the underlying mechanisms of action.

## Author contributions

Mariska Mantione made a substantial contribution to the design of the work, the acquisition and interpretation of the work, drafted the work, approved the final version to be published and agreed to be accountable for all aspects of the work.

Martijn Figee made a substantial contribution to the interpretation of the work, revised the work critically for important intellectual content, approved the final version to be published and agreed to be accountable for all aspects of the work.

Damiaan Denys made a substantial contribution to the design and interpretation of the work, revised the work for important intellectual content, approved the final version to be published and agreed to be accountable for all aspects of the work.

## Conflict of interest statement

The authors declare that the research was conducted in the absence of any commercial or financial relationships that could be construed as a potential conflict of interest.
